# Dexmedetomidine as a neuraxial adjuvant for prevention of perioperative shivering: Meta-analysis of randomized controlled trials

**DOI:** 10.1371/journal.pone.0183154

**Published:** 2017-08-22

**Authors:** Jian Zhang, Xuena Zhang, Hui Wang, Haibin Zhou, Tian Tian, Anshi Wu

**Affiliations:** 1 Department of Anesthesiology, Beijing Chaoyang Hospital of Capital Medical University, Beijing, China; 2 Department of Anesthesiology, Ji Shui Tan Hospital and Fourth Medical College of Peking University, Beijing, China; 3 Department of Anesthesiology, Beijing Xuanwu Hospital of Capital Medical University, Beijing, China; Southeast University Zhongda Hospital, CHINA

## Abstract

**Background:**

Dexmedetomidine, a highly selective α2-adrenoceptor agonist, has been investigated for anti-shivering effects in some trials. This current meta-analysis was conducted to evaluate the effectiveness of dexmedetomidine as a neuraxial adjuvant in preventing perioperative shivering.

**Methods:**

This systematic review and meta-analysis was registered in PROSPERO [www.crd.york.ac.uk/PROSPERO] with the unique identification number CRD42017055991. The electronic databases PubMed, Embase, Cochrane Central Register of Controlled Trials (CENTRAL) were searched to select high-quality randomized controlled trials (RCTs) that evaluated the anti-shivering efficacy for neuraxial application dexmedetomidine as local anesthetic adjuvant. Effects were summarized using pooled risk ratios (RRs), weighed mean differences (MDs), or standardized mean differences (SMDs) and corresponding 95% confidence intervals (Cls) with random effect model. Heterogeneity assessment, sensitivity analysis, and publication bias were performed. The primary outcome was perioperative shivering.

**Results:**

A total of 1760 patients from 24 studies were included in this meta-analysis. Compared with the placebo, dexmedetomidine reduced the incidence of perioperative shivering (RR: 0.34; 95% Cl: 0.21 to 0.55; P < 0.00001), with a maximum effective dose of 5μg via subarachnoid space injection (RR: 0.55; 95% CI: 0.32 to 0.92; P = 0.02), especially in cesarean section (RR: 0.20; 95% CI: 0.09 to 0.45; P = 0.0001). Dexmedetomidine also could improve the characteristics of the block, with an increase only in the incidence of bradycardia (RR: 2.11; 95% CI: 1.23 to 3.60; P = 0.006). No significant difference could be found compared dexmedetomidine with other adjuvants, except morphine.

**Conclusions:**

This meta-analysis shows that dexmedetomidine as a neuraxial adjuvant had statistically significant efficacy on prevention of perioperative shivering. Moreover, dexmedetomidine could improve the characteristics of the block. However, the potential induction of bradycardia should be taken seriously.

## Introduction

Neuraxial anesthesia is the most commonly employed for lower abdominal, perineum and lower limb surgery. It has the advantages of easy administration technique, less adverse effects, cost-effectiveness and the patient remaining conscious throughout the procedure, compared with general anesthesia. One of the most common complications after neuraxial anesthesia is perioperative shivering with reported incidences in the range of 36% to 85% [1]. The mechanism of shivering under neuraxial anesthesia is attributed to the loss of thermoregulatory vasoconstriction below the blockage, which could inhibit tonic vasoconstriction and redistribute core heat [2]; risk factors for hypothermia include ageing, the height of the applied block [3], and the temperature of the operation room and IV solutions.

Shivering is defined as an involuntary rhythmic activity of skeletal muscles, and it can bring about a feeling of discomfort and phobia in awake patients [4], increase the sensation of cold and wound pain and delay wound healing [5]. Additionally, it increases oxygen consumption, carbon dioxide production, as well as catecholamine secretion, with a subsequent increase in basal metabolic rate, which may cause severe adverse effects in patients with cardiopulmonary insufficiency [4, 6]. Some studies have proven that several pharmacological agents, such as ketamine, nefopam, clonidine, pethidine, tramadol, and granisetron [7], are useful for the prevention of shivering, but they are limited in their administration to clinical practice due to their unsatisfactory therapeutic effect [8] and side effects [9, 10].

Dexmedetomidine is a highly selective α2-adrenoceptor agonist that binds to a transmembrane G protein-binding receptor. However, US Food and Drug Administration (FDA) have not approved dexmedetomidine for neuraxial administration. Pre-clinic evidence showed that dexmedetomidine, used as a local anesthetic adjuvant for intravertebral anesthesia, can shorten the onset time of the block [11], decrease postoperative pain intensity [12], prolong the duration of the block [13] and reduce the requirement of the analgesics [14]. Most importantly, it can increase vasodilation and the thresholds of shivering, and inhibit central thermoregulation [15]. Clinical research has focused on the effect of dexmedetomidine on perioperative shivering, but with controversial results. Hence, we here conducted a meta-analysis to assess the effectiveness of dexmedetomidine, used as a neuraxial adjuvant, on the prevention of perioperative shivering.

## Methods

### Systematic search and strategy

This systematic review was performed in accordance with the Preferred Reporting Items for Systematic Reviews and Meta-analyses (PRISMA) statement [16, 17]. The protocol was registered in PROSPERO (www.crd.york.ac.uk/PROSPERO) with the unique identification number CRD 42017055991.

The electronic databases PubMed, Embase, and Cochrane Central Register of Controlled Trials (CENTRAL) were searched up to January 15, 2017, without language limitations. We also searched the reference lists of the included studies and grey literature using the System for Information on Grey Literature in Europe (SIGLE) database to identify potential RCTs. The search strategy consisted of a combination of free text words and Medical Subject Headings (MeSH) terms. The full details of the search strategy are provided in the Appendix. The search equation for PubMed was adapted for each database.

### Eligibility criteria

Studies were included in the systematic review if they satisfied all the following pre-established criteria: (1) randomized controlled trial; (2) Jadad scale >3; (3) neuraxial dexmedetomidine was delivered via any intravertebral routes, such as epidural, intrathecal, and caudal route in adults and children of any sex undergoing elective surgical procedure; (4) the reported presence or absence of shivering.

Exclusion criteria: We excluded studies if they (1) were duplicate publications, reviews, abstracts from conferences, letters to the editor, or animal studies, (2) included patients with a history of allergy to dexmedetomidine, or other contradictions for dexmedetomidine, and (3) did not report the specific result of shivering.

### Data extraction and risk of bias assessment

Two reviewers (JZ and XZ) independently assessed the studies for compliance with the eligibility criteria. Any discrepancy was resolved by consultation with a third reviewer (AW). The PRISMA flow diagram was used to summarize the processes of study selection.

Extracted data included the name of the first author, publication year, study design, participants’ demographic characteristics, ASA physical status, type of surgery, dose and route of dexmedetomidine administration, and number of shivering cases. Two reviewers (JZ and HW) did the extraction of all data mentioned above, while another reviewer (HZ) checked the extracted data.

Two authors (JZ and HZ) evaluated the overall risk of bias in individual studies according to the guidelines recommended by the Cochrane Collaboration with regard to the adequacy of randomization, concealment of allocation, blinding (of patients, healthcare providers, and outcome assessors), incomplete outcome data, selective outcome reporting, and other sources of bias. Each parameter was classified into “low”, “high”, or “unclear”.

### Assessment of study quality

An evaluation of the studies quality was performed by 2 reviewers (JZ and TT) by using a 5point Jadad scale[18]. The main categories consisted of the following 5 items: ‘‘Was the study described as randomized?”, ‘‘Was the method used to generate the sequence of randomization described and appropriate?”, ‘‘Was the study described as double-blind?”, ‘‘Was the method of double-blinding described and appropriate?”, and ‘‘Was there a description of withdrawals and drop-outs?”. A score of below 4 was considered a low methodological quality.

### Statistical analysis

For binary variables, the pooled risk ratios (RRs) and 95% confidence intervals (95% CIs) were calculated. Continuous data were assessed by pooled weighted mean difference (MD) or pooled standard mean difference (SMD). SMD was calculated for the time to rescue analgesia because of different units. The overall effect was assessed by Z test using a random effects model (Inverse Variance method) [19] and statistical significance was determined when the 95% CIs did not include the value of 1.0 for the RR or 0 for the MD or SMD. The statistics of I^2^ and corresponding 95% Cl were used to measure heterogeneity (DerSimonian-Laird method) [20]. For trials assessing different doses, the groups were combined to create a single pair-wise comparison [21].

Subgroup analyses were performed on the doses and routes administered, as well as for the type of surgery. Sensitivity analyses were performed to test the reliability of the results by removing each study individually and changing effects model of the statistical method (fixed-effect model [Mantel-Haenszel method] vs. random-effect model [Inverse Variance method]). Potential publication bias was evaluated using Egger’s regression test. In addition to assess the possibility of small study bias, we conducted a trim and fill analysis, which was a sensitivity analysis for potential publication bias with Stata (Version 13.0.; Stata Corp, TX, USA), and statistical analyses were accomplished using Review Manager (Rev Man) (Version 5.3.; The Cochrane Collaboration, Oxford, UK).

## Results

### Study selection

**Fig 1:**
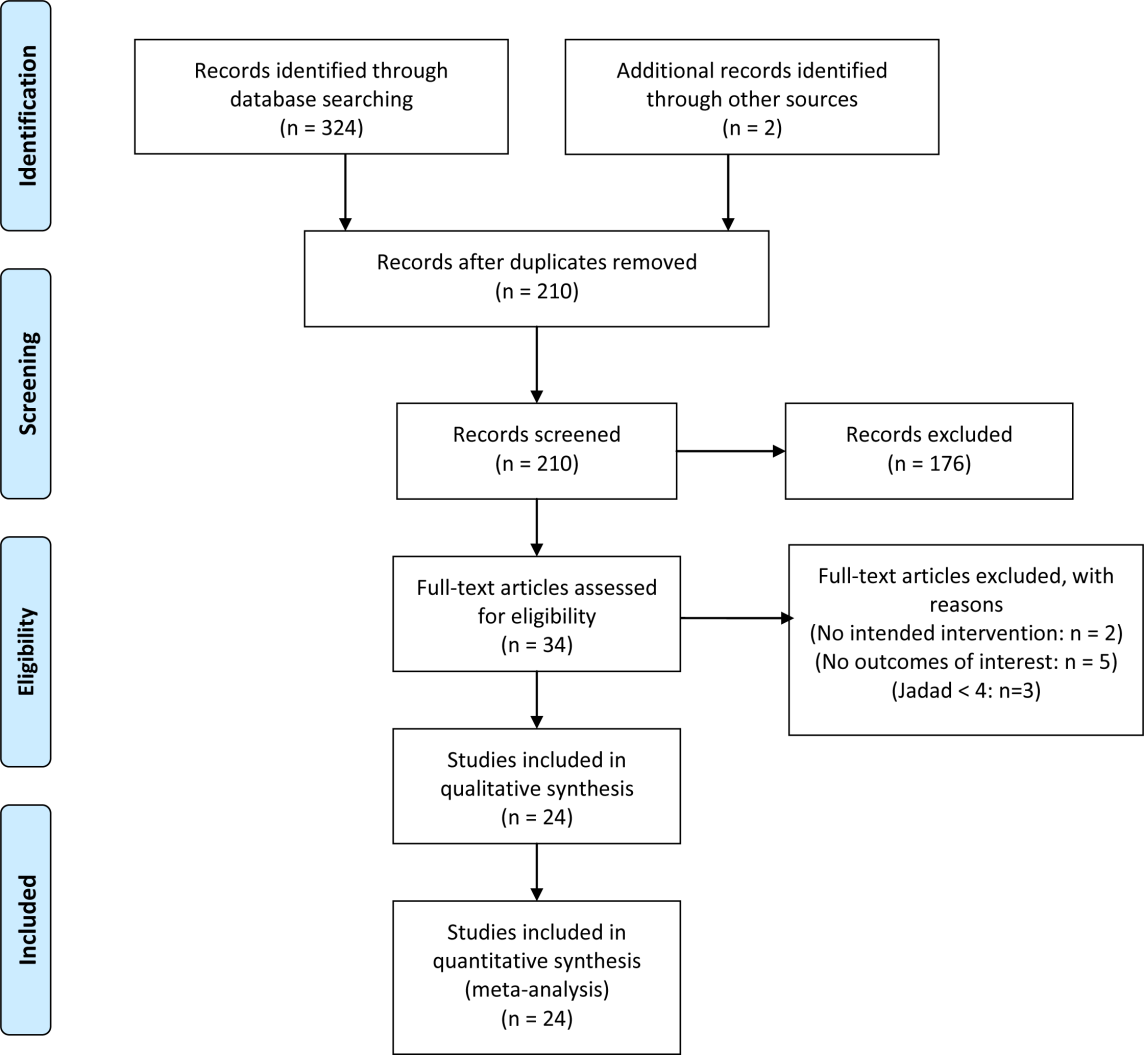
Flow diagram of the inclusion and exclusion processes.

Systematically search of PubMed, Embase, CENTRAL, SIGLE and reference lists generated 324 articles, and we identified two additional citations through other sources. Of these, 116 were duplications and were excluded. Then, after retrieval and review of the articles’ abstracts, 176 studies were excluded based on the title and abstract. The remaining 34 studies were examined in detail. A further 10 studies were then excluded because of Jadad scale < 4, a lack of intended intervention and outcomes of interest. Finally, 24 studies [22–45] fulfilled the criteria for systematic review and meta-analysis. The study selection processes are shown in Fig 1.

### Study characteristics

Fifteen of the included trials reported on the effectiveness of dexmedetomidine on the prevention of perioperative shivering compared with placebo, 11 studies [22–30, 44, 45] administered via spinal route, and 4[36–39] researches via epidural route. Patients investigated in 5 trials [25, 38, 39, 44, 45] were nearly full term parturients selected for cesarean section. Five studies [22, 28, 31–33] compared different doses of dexmedetomidine via subarachnoid administration. Other control adjuvants included clonidine [40, 42, 44], fentanyl [24, 34, 39, 43–45], morphine [25], midazolam [23], buprenorphine [35], and butorphanol [41].

Of the included 15 studies, twelve studies showed the characteristics of the block, including the onset of sensory block [22, 23, 25, 26, 28, 30, 38, 39], the onset of motor block [22, 23, 25, 26, 28, 44, 45], the duration of the sensory block [22, 24–28, 30, 39, 44, 45], the duration of motor block [22, 24–28, 38, 44, 45], and the time to rescue analgesia [22, 23, 25–28, 30, 38, 44, 45]. Side effects were reported in 15 studies, comprising neurological complications [24, 25, 28, 30], respiratory depression [22, 24–26, 28–30, 38, 44, 45], bradycardia [22–30, 36, 38, 44, 45], hypotension [22–27, 29, 30, 36, 38, 44, 45], nausea/vomiting [22–30, 36–39, 44, 45]. None of the studies reported mortality and major cardiovascular complications, such as non-fatal myocardial infarction, stroke, or cardiac arrest. S1 Table shows the characteristics of all included studies.

### Risk of bias within studies

**Fig 2:**
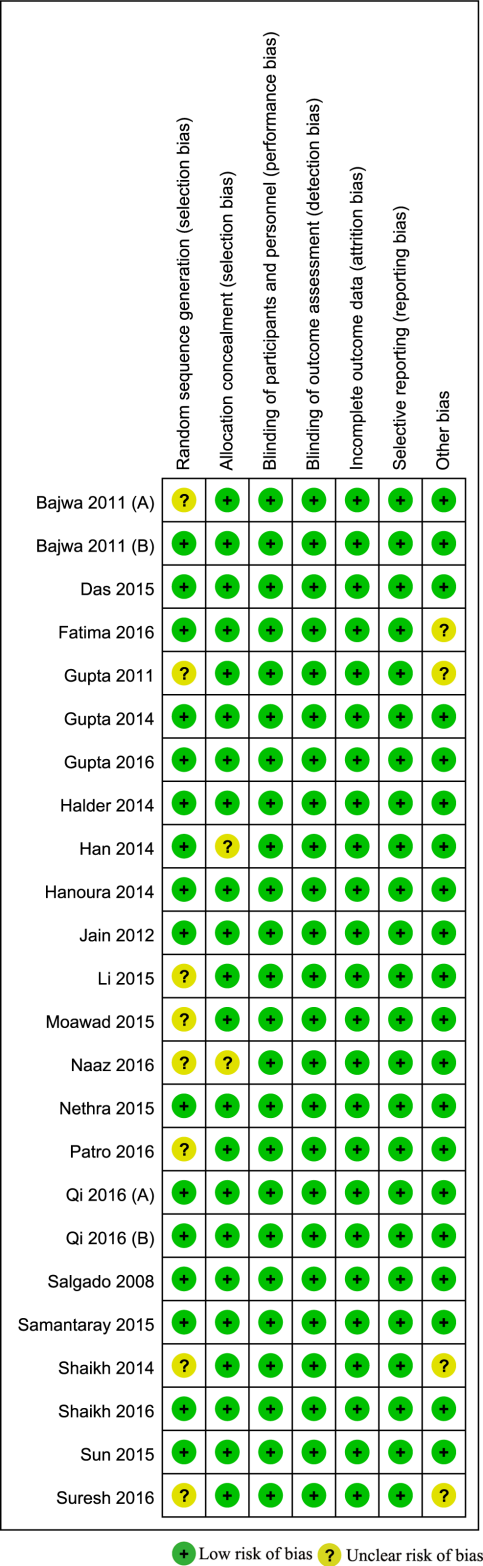
Risk of bias assessment of the included studies.

All trials were described as having a randomized trial design, while 8 [22, 26, 28–30, 34, 43, 44] of 24 studies did not describe detailed information about random sequence generation. Two studies [28, 39] did not describe the methods of allocation concealment, and all reports were double-blinded. No incomplete outcomes (attrition bias) and selective reporting (reporting bias) were reported in any of the trials. Four studies [22, 30, 34, 41] did not describe detailed information about the time of surgery, and thus some biases were unclear. An overview of the risk of bias is given in Fig 2.

### Results of meta-analysis

#### Dexmedetomidine versus placebo

**Fig 3:**
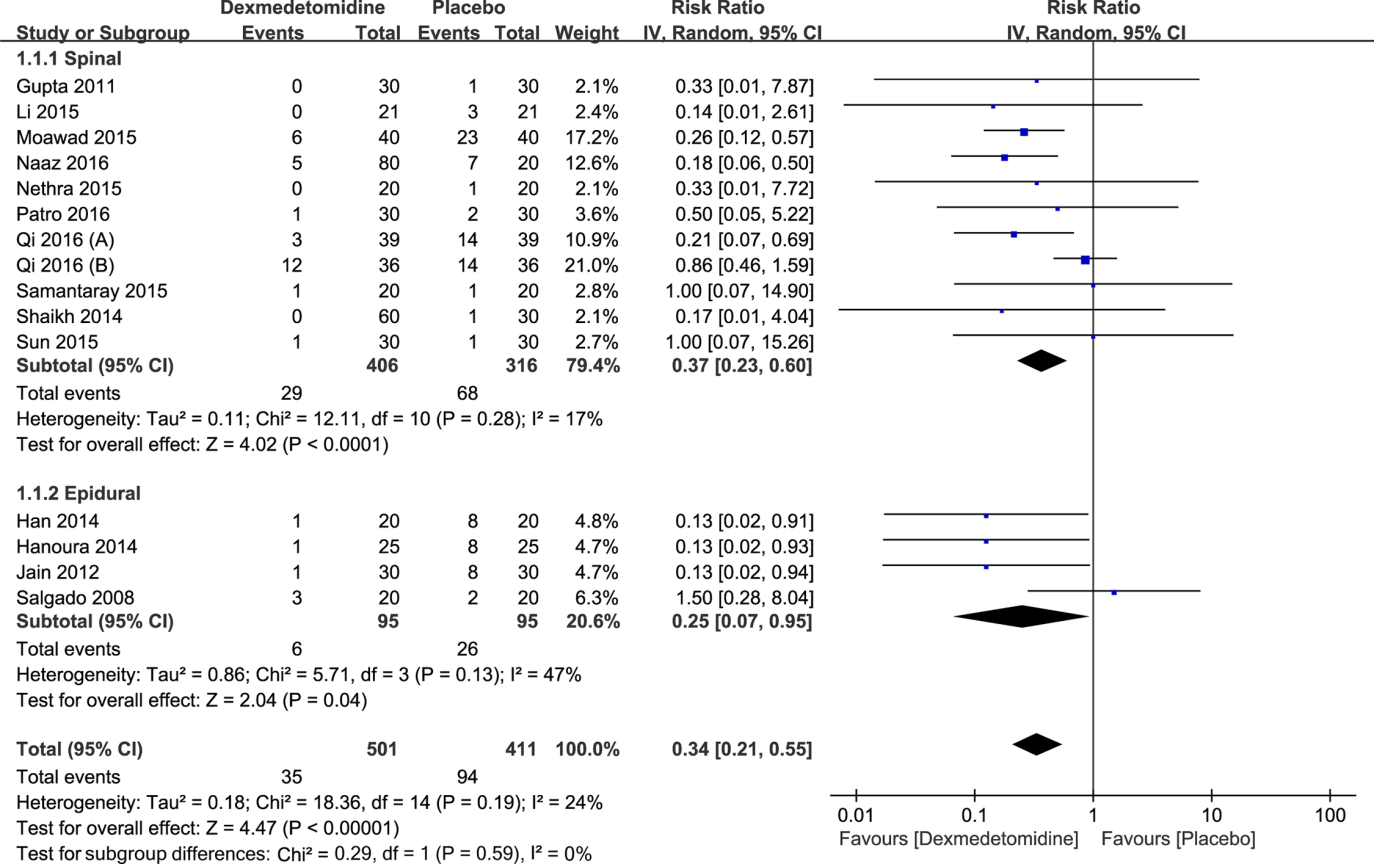
Results of subgroup analysis of the incidence of perioperative shivering by routes of dexmedetomidine administration.

Shivering. Fifteen [22–30, 36–39, 44, 45] studies including 912 participants assessed the effectiveness of dexmedetomidine compared with placebo on the prevention of perioperative shivering in neuraxial anesthesia. As shown in Fig 3, dexmedetomidine was significantly more effective than placebo for the prevention of perioperative shivering (RR: 0.34; 95% CI: 0.21 to 0.55; I^2^ = 24%; 95% Cl: 0% to 59%).

The funnel plot and egger regression test did not suggest any publication bias (P = 0.311). The trim and fill analysis did not show any evidence of asymmetry. Sensitivity analysis of the shivering by removing each study individually and changing effects model of the statistical method did not alter the finding above (S1 Fig).

Subgroup analyses were carried out to evaluate the factors that affected perioperative shivering.

Routes of administration. The subgroup analysis of the incidence of perioperative shivering, including 912 participants from fifteen studies, was performed by routes of dexmedetomidine administration, and regardless of the route of dexmedetomidine administration, comprising subarachnoid space injection (RR: 0.37; 95% CI: 0.23 to 0.60; I^2^ = 17%; 95% Cl: 0% to 58%) and epidural space injection (RR: 0.25; 95% CI: 0.07 to 0.95; I^2^ = 47%; 95% Cl: 0% to 83%), the incidence of shivering was lower in the dexmedetomidine group (Fig 3).

**Fig 4:**
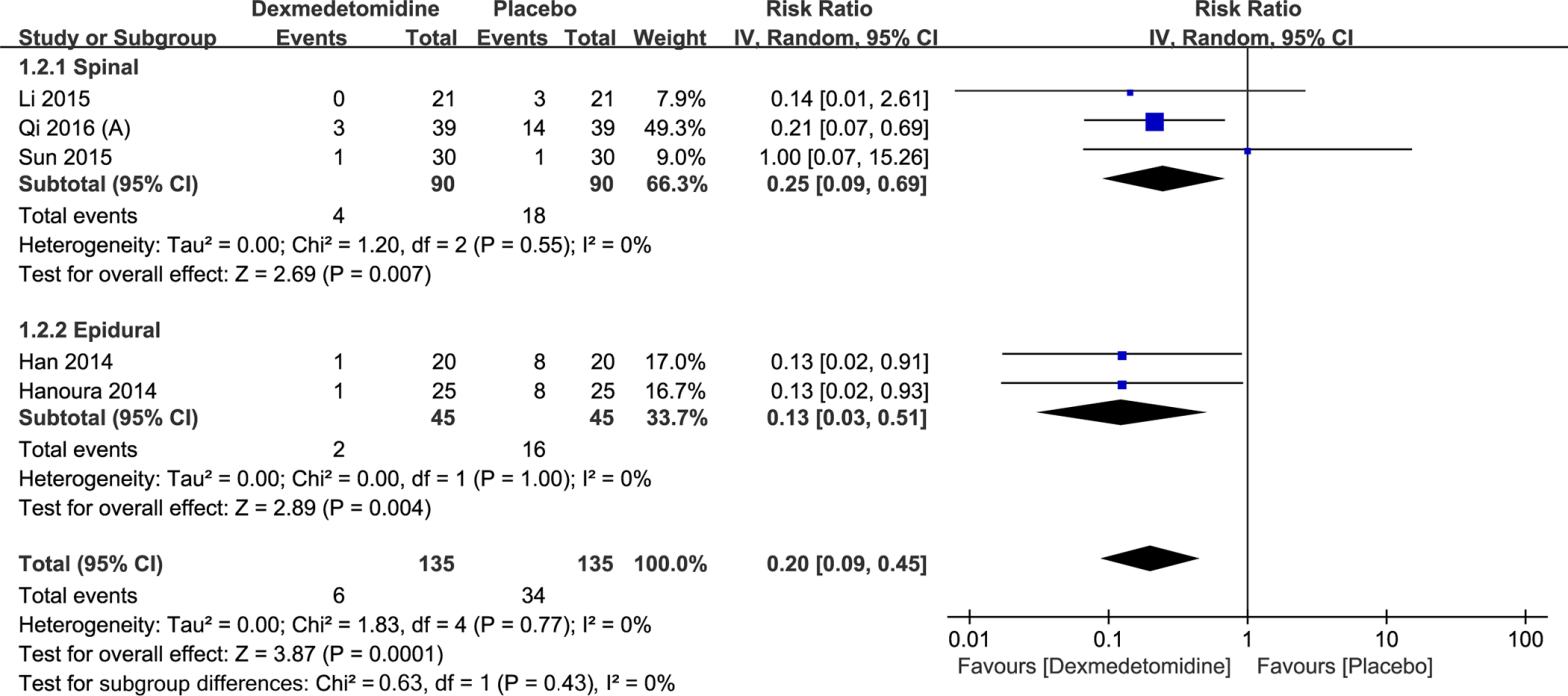
Results of subgroup analysis of the incidence of perioperative shivering in cesarean section.

Cesarean section. This subgroup analysis involved 270 participants from five studies. Dexmedetomidine significantly reduced the incidence of shivering in cesarean section (RR: 0.20; 95% CI: 0.09 to 0.45; I^2^ = 0%; 95% Cl: 0% to 79%), spinal administration (RR: 0.25; 95% CI: 0.09 to 0.69; I^2^ = 0%; 95% Cl: 0% to 90%) or epidural administration (RR: 0.13; 95% CI: 0.03 to 0.51; I^2^ = 0%) (Fig 4).

**Fig 5:**
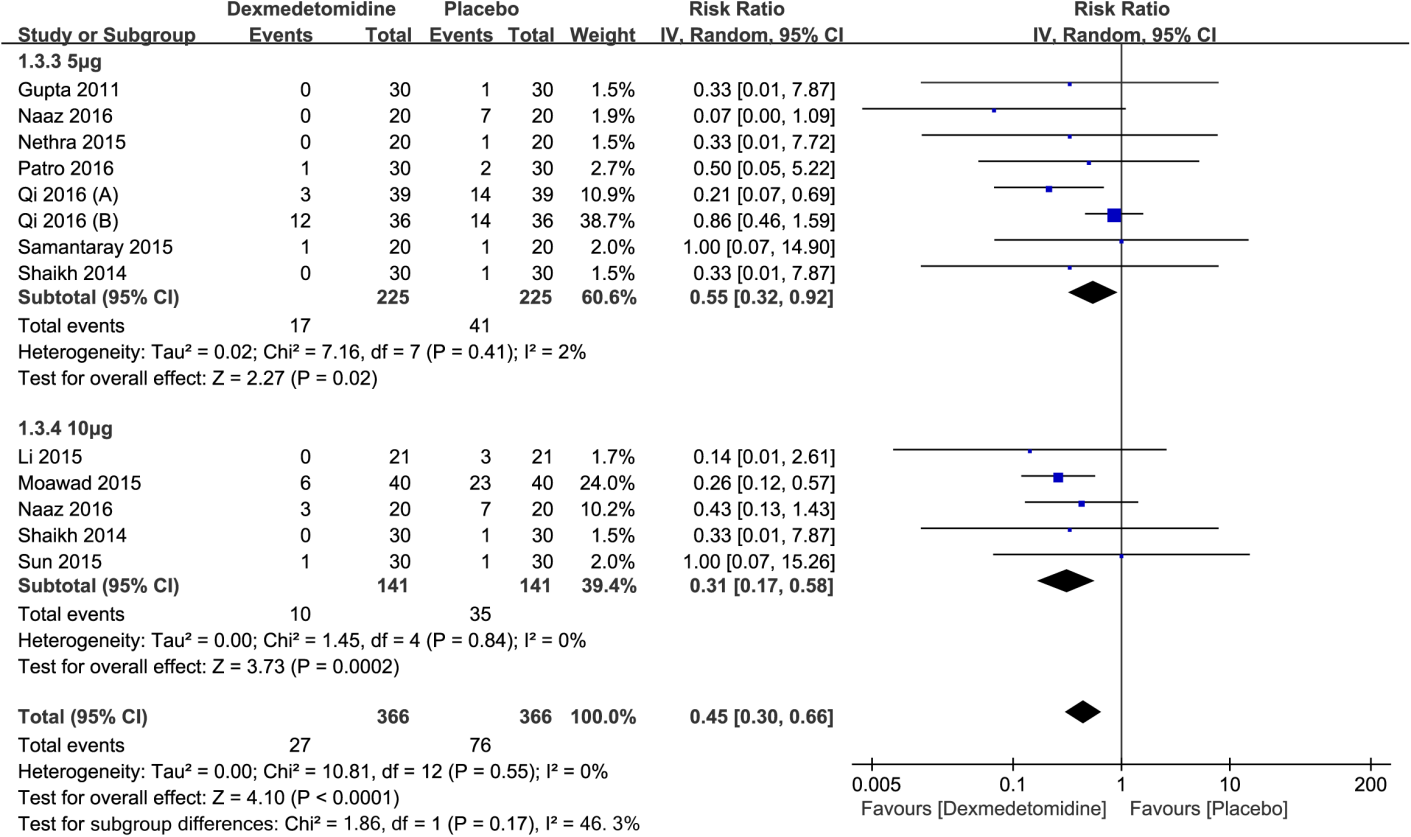
Results of subgroup analysis of the incidence of perioperative shivering by doses of spinal dexmedetomidine.

Dose of dexmedetomidine. This subgroup analysis involved 732 participants from eleven studies. It was also carried out to evaluate the different dose of dexmedetomidine that affected perioperative shivering. Injected into the subarachnoid space, both dexmedetomidine 5μg (RR: 0.55; 95% CI: 0.32 to 0.92; I^2^ = 2%; 95% Cl: 0% to 68%) and dexmedetomidine 10μg (RR: 0.31; 95% CI: 0.17 to 0.58; I^2^ = 0%; 95% Cl: 0% to 79%) were significantly more effective than placebo for the prevention of perioperative shivering (RR: 0.45; 95% CI: 0.30 to 0.66; I^2^ = 0%; 95% Cl: 0% to 57%) (Fig 5).

The funnel plot and egger regression test did not suggest any publication bias among three subgroup analyses above (P = 0.311, 0.810, 0.284). The trim and fill analysis did not show any evidence of asymmetry. Sensitivity analysis of the shivering by removing each study individually did not alter the finding above (S2 Fig).

Characteristics of the block. The characteristics of spinal blockade are summarized in Table 1. The time of onset to block was significantly shorter in the dexmedetomidine group compared with the placebo group, including onset of sensory block (MD: -0.87 minutes; 95% CI: -1.38 to -0.36; P = 0.0009) and onset of motor block (MD: -1.08 minutes; 95% CI: -1.38 to -0.79; P < 0.00001). Dexmedetomidine could prolong the duration of the block, which was also statistically significant as compared with placebo, the duration of the sensory block (MD: 100.39 minutes; 95% CI: 69.08 to 131.69; P < 0.00001), the duration of the motor block (MD: 59.61 minutes; 95% CI: 32.91 to 86.32; P < 0.0001). Additionally, the time to rescue analgesia was significantly longer in the dexmedetomidine group (SMD: 4.63; 95% CI: 3.27 to 5.98; P < 0.00001). Sensitivity analysis of characteristics of the block by removing each study individually did not alter the finding above (S3 Fig).

**Table 1:** Comparison of characteristics of spinal blockade between dexmedetomidine and placebo.

Characteristics of spinal blockade	Number of studies	Random-effect model MD (95% CI) (min) or SMD (95% CI)	Fixed-effect model MD (95% CI) (min) or SMD (95% CI)	References
**Onset of sensory block**	8	-0.87 (-1.38 to -0.36)	-1.10 (-1.23 to -0.98)	[22, 23, 25, 26, 28, 30, 38, 39]
Spinal route	6	-0.65 (-1.13 to -0.17)	-1.09 (-1.22 to -0.97)	[22, 23, 25, 26, 28, 30]
Epidural route	2	-2.45 (-6.57 to 1.66)	-1.55 (-2.37 to -0.72)	[38, 39]
**Onset of motor block**	7	-1.08 [-1.38, -0.79]	-1.20 [-1.32, -1.07]	[22, 23, 25, 26, 28, 44, 45]
Spinal route	7	-1.08 [-1.38, -0.79]	-1.20 [-1.32, -1.07]	[22, 23, 25, 26, 28, 44, 45]
Epidural route	-	-	-	-
**Duration of sensory block**	10	100.39 [69.08, 131.69]	87.14 [84.71, 89.57]	[22, 24–28, 30, 39, 44, 45]
Spinal route	9	96.55 [63.77, 129.33]	87.01 [84.57, 89.44]	[22, 24–28, 30, 44, 45]
Epidural route	1	142.00 [91.86, 192.14]	142.00 [91.86, 192.14]	[39]
**Duration of motor block**	9	59.61 [32.91, 86.32]	76.24 [73.28, 79.21]	[22, 24–28, 38, 44, 45]
Spinal route	8	65.72 [38.63, 92.81]	78.71 [75.69, 81.74]	[22, 24–28, 44, 45]
Epidural route	1	11.10 [-4.43, 26.63]	11.10 [-4.43, 26.63]	[38]
**Time to rescue analgesia**	10	4.63 [3.27, 5.98]	3.06 [2.78, 3.35]	[22, 23, 25–28, 30, 38, 44, 45]
Spinal route	9	4.23 [2.91, 5.54]	2.93 [2.64, 3.21]	[22, 23, 25–28, 30, 44, 45]
Epidural route	1	8.30 [6.52, 10.09]	8.30 [6.52, 10.09]	[38]

**Abbreviations:** CI, confidence interval; MD, weighted mean difference (min); SMD, standard mean difference.

Adverse effects. The meta-analysis showed that dexmedetomidine increased the probability of bradycardia (RR: 2.11; 95% CI: 1.23 to 3.60; I^2^ = 0%; 95% Cl: 0% to 58%), but had no significant effect with regard to the rates of other common adverse effects, such as hypotension (RR: 1.24; 95% CI: 0.90 to 1.71; I^2^ = 0%; 95% Cl: 0% to 60%), nausea/vomiting (RR: 0.84; 95% CI: 0.51 to 1.38; I^2^ = 0%; 95% Cl: 0% to 55%), respiratory depression (RR: 4.41; 95% CI: 0.26 to 73.32; I^2^ = NA) (Table 2). Sensitivity analysis of adverse effects by removing each study individually did not alter the finding above (S4 Fig).

**Table 2:** Comparison of incidences of adverse effects between dexmedetomidine and placebo.

Adverse effects	Number of studies	Incidence of adverse effects/total number of patients		Fixed-effect model RR (95% CI)	Random-effect model RR (95% CI)
		Dexmedetomidine	Placebo		
**Bradycardia**	12	48/451	16/361	2.21 (1.31 to 3.72)	2.11 [1.23, 3.60]
Spinal route	10	39/406	13/316	2.09 (1.15 to 3.78)	1.95 [1.05, 3.60]
Epidural route	2	9/45	3/45	2.71 (0.90 to 8.18)	2.71 [0.90, 8.12]
**Hypotension**	11	60/371	45/341	1.30 (0.93 to 1.80)	1.24 (0.90 to 1.71)
Spinal route	9	45/326	32/296	1.35 (0.91 to 2.01)	1.30 (0.87 to 1.94)
Epidural route	2	15/45	13/45	1.15 (0.66 to 2.02)	1.13 (0.66 to 1.95)
**Nausea/Vomiting**	14	30/501	33/411	0.88 (0.55 to 1.39)	0.84 (0.51 to 1.38)
Spinal route	10	19/406	22/316	0.81 (0.45 to 1.45)	0.81 (0.44 to 1.49)
Epidural route	4	11/95	11/95	1.00 (0.47 to 2.13)	1.02 (0.33 to 3.19)
**Respiratory depression**	10	8/391	0/301	4.41 [0.26, 73.32]	4.41 [0.26, 73.32]
Spinal route	9	8/366	0/276	4.41 [0.26, 73.32]	4.41 [0.26, 73.32]
Epidural route	1	0/25	0/25	NA	NA
**Neurological complications**	4	1/185	0/125	3.00 [0.13, 71.28]	3.00 [0.13, 71.28]
Spinal route	4	1/185	0/125	3.00 [0.13, 71.28]	3.00 [0.13, 71.28]
Epidural route	-	-	-	-	-

**Abbreviations:** CI, confidence interval; RR, relative risk; NA, not applicable.

#### Dexmedetomidine versus dexmedetomidine

**Fig 6:**
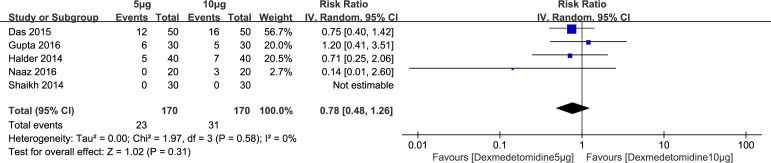
Results of subgroup analysis of the incidence of perioperative shivering compared dexmedetomidine 5μg with dexmedetomidine 10μg.

Five studies including 340 participants compared dexmedetomidine 5μg with dexmedetomidine 10μg. When dexmedetomidine administered by spinal injection, the statistical analysis failed to achieve significance (RR: 0.78; 95% CI: 0.48 to 1.26; I^2^ = 0%; 95% Cl: 0% to 85%) (Fig 6). The funnel plot and egger regression test did not suggest any publication bias (P = 0.453). The trim and fill analysis did not show any evidence of asymmetry. Sensitivity analysis of the shivering by removing each study individually and changing effects model of the statistical method did not alter the finding above.

#### Dexmedetomidine versus other adjuvants

Twelve studies [23–25, 34, 35, 39–45] involving 765 patients compared the efficacy of dexmedetomidine with other adjuvants on perioperative shivering. No significant difference could be found between dexmedetomidine and other adjuvants, including clonidine, fentanyl, midazolam, buprenorphine, butorphanol, except morphine (RR: 0.26; 95% CI: 0.08 to 0.84; I^2^ = NA) (S2 Table). Of 2 trials exploring neurological complications, only 1 study [28] reported that neurological complication occurred in one of 36 patients in each dexmedetomidine group and fentanyl group.

## Discussion

The present meta-analysis, which included 24 studies, revealed that dexmedetomidine as a neuraxial adjuvant could significantly reduce the incidence of perioperative shivering compared with placebo. Both spinal and epidural routes of dexmedetomidine administration could demonstrate the beneficial anti-shivering effect, with a maximum effective dose of 5μg via subarachnoid space injection. With regard to obstetric patients selected for cesarean section, dexmedetomidine could effectively prevent perioperative shivering. Furthermore, dexmedetomidine also enhanced the characteristics of the block. In terms of adverse effects, dexmedetomidine only increased the probability of bradycardia. However, dexmedetomidine failed to show superiority over other anti-shivering agents in the prevention of perioperative shivering, except morphine.

Several reviews [5, 46] conclude the potential role ofα2 adrenoreceptor (α2-AR) agonist for perioperative shivering control. Dexmedetomidine, a highly lipophilic anesthetic [47], demonstrates almost 8- 10times higher affinity to α2-ARs than clonidine [48]. When administered as a neuraxial adjuvant, dexmedetomidine can quickly bind to dorsal horn of the spinal cord α2-ARs, subsequently to inhibit the spontaneous firing rate of neurons [49] and sympathetic tone [29]. However, mechanisms of hypothermia and shivering may differ in parturients. The parturients can occur shivering even after normal delivery[46], and the anti-shivering mechanism of dexmedetomidine can be explained by the attenuation of hyperadrenergic response to perioperative stress[44].

The pooled results from our meta-analysis showed that both spinal and epidural dexmedetomidine is an option for anti-shivering compared with placebo. Nevertheless, the previous meta-analyses[5] failed to assess the effectiveness of spinal dexmedetomidine on the prevention of perioperative shivering because of the limited number of included studies. Our further subgroup analysis, based on the data of the 11 included studies, revealed that both 5μg and 10μg doses of spinal dexmedetomidine could effectively prevent perioperative shivering. However, spinal dexmedetomidine 10μg failed to show superiority over dexmedetomidine 5μg in the prevention of perioperative shivering. Therefore, we concluded that the maximum effective dose of spinal dexmedetomidine was 5μg.

The pooled results from our meta-analysis showed that dexmedetomidine used as a local anesthetic adjuvant for intravertebral anesthesia could improve the characteristics of the block, such as shortening the onset time of the block, and prolonging the duration of the block and rescue analgesia time. These findings were similar to previous numerous studies [50, 51]. Subgroup analysis of different routes of dexmedetomidine administration confirmed the conclusions, except onset of sensory block and duration of motor block due to the limited trials. The mechanisms were related to hyperpolarization of post-synaptic dorsal horn neurons [52], α2-adrenoceptor agonists to motor neurons in the dorsal horn [53], and upregulation of the adrenergic receptor subtypes on the dorsal horn and the lumbar dorsal root ganglia [54].

The pooled results from our meta-analysis showed that dexmedetomidine made induced bradycardia in more patients compared with placebo, which was in agreement with previous studies[12–14]. No evidence indicated any increased risk of other adverse events, such as hypotension, nausea/vomiting. We also carried out subgroup analysis for type of dexmedetomidine administration to consolidate results, and only epidural dexmedetomidine missed the significantly statistical difference of bradycardia. The results above could be attributed to the inhibition of endogenous catecholamines [54] and the depressurization effect of spinal anesthesia [55]. Since our meta-analysis failed to allow any conclusion about the neurotoxic safety of dexmedetomidine, one had to consider other sources of evidence before exposing the spinal cord to a substance that was not approved for spinal application in any country in the world. In this context, one had to consider that at least experimentally several animal studies [56, 57] had demonstrated that dexmedetomidine could cause neurotoxic effects, which should be taken seriously. Although we observed an 8-fold higher frequency of respiratory depression in one study [28], this did not result in a significant difference. However, this may be at least a signal that under high-dose conditions the administration of spinal dexmedetomidine may result in respiratory depression.

A previous meta-analysis[5] had shown that there were no significant differences between dexmedetomidine and other agents, which were similar to our findings. Subgroup analysis of different routes of dexmedetomidine administration confirmed our results. Nevertheless, few studies of our meta-analysis comparing dexmedetomidine with other adjuvants were assessed and had a high risk of bias. Therefore, the results needed to be further confirmed.

It is meaningful to shed light on the effectiveness of dexmedetomidine as a neuraxial adjuvant on prevention of perioperative shivering by means of a meta-analysis of high-quality RCTs. Most of the included studies were well designed and assessed as having a low risk of bias; sensitivity analysis was performed by removing each study individually and changing effects model of the statistical method, and thus the accuracy of the outcomes is verified.

Our study has several limitations. First, with some subgroup meta-analyses of small numbers, we failed to really examine publication bias and the confidence intervals of the heterogeneity were very wide, hence we were extremely uncertain about the validity of the estimates. Second, all the participants were adults, so we failed to evaluate whether dexmedetomidine was effective for preventing shivering in children via caudal administration. Furthermore, high risk factors of hypothermia, such as room temperature and the temperature of the IV solutions, could not be monitored throughout the literature reports, and therefore we could not include these as evaluation items. Finally, few studies have compared the efficacy of dexmedetomidine with other drugs on perioperative shivering; thus, we failed to conclude the superiority of dexmedetomidine and evaluate adverse effects, such as neurological complication; it calls for more RCTs to address this question.

## Conclusions

In conclusion, this current meta-analysis suggested that dexmedetomidine as a neuraxial adjuvant had statistically significant efficacy on prevention of perioperative shivering, with a maximum effective dose of 5μg via spinal administration. Dexmedetomidine also could significantly reduce the incidence of shivering in cesarean section. Moreover, dexmedetomidine could improve the characteristics of the block. However, when dexmedetomidine is used as a neuraxial adjuvant, the potential development of bradycardia should be considered.

## Supporting information

S1 Fig A: funnel plot for publication bias for incidence of shivering, B: sensitivity analysis for the shivering by removing each study individually.
(TIF)

S2 Fig A: funnel plot for publication bias for routes of administration, B: funnel plot for publication bias for cesarean section, C: funnel plot for publication bias for different doses of dexmedetomidine, D: sensitivity analysis for routes of administration, E: sensitivity analysis for cesarean section, F: sensitivity analysis for different doses of dexmedetomidine.
(TIF)

S3 Fig A: sensitivity analysis for onset of sensory block, B: sensitivity analysis for onset of motor block, C: sensitivity analysis for duration of sensory block, D: sensitivity analysis for duration of motor block, E: sensitivity analysis for time to rescue analgesia.
(TIF)

S4 Fig A: sensitivity analysis for bradycardia, B: sensitivity analysis for hypotension, C: sensitivity analysis for nausea/vomiting.
(TIF)

S1 Table Characteristics of studies included in the present systematic review and meta-analysis. Abbreviations: ASA, American Society of Anesthesiologists physical status; SA, spinal anesthesia; EA, epidural anesthesia; L, Lumbar; DEX, dexmedetomidine.
(DOCX)

S2 Table Comparison of incidences of shivering between dexmedetomidine and other adjuvants. Abbreviations: CI, confidence interval; RR, relative risk; NA, not applicable.
(DOCX)

S1 File PRISMA checklist. (DOC)

S1 Appendix PubMed search strategy. (TIF)
