# Supplementary figures and images for: Dexmedetomidine as a neuraxial adjuvant for prevention of perioperative shivering: Meta-analysis of randomized controlled trials
Source: PLoS One. 2017 Aug 22;12(8):e0183154. doi: 10.1371/journal.pone.0183154 (PMC5567500; doi:10.1371/journal.pone.0183154)

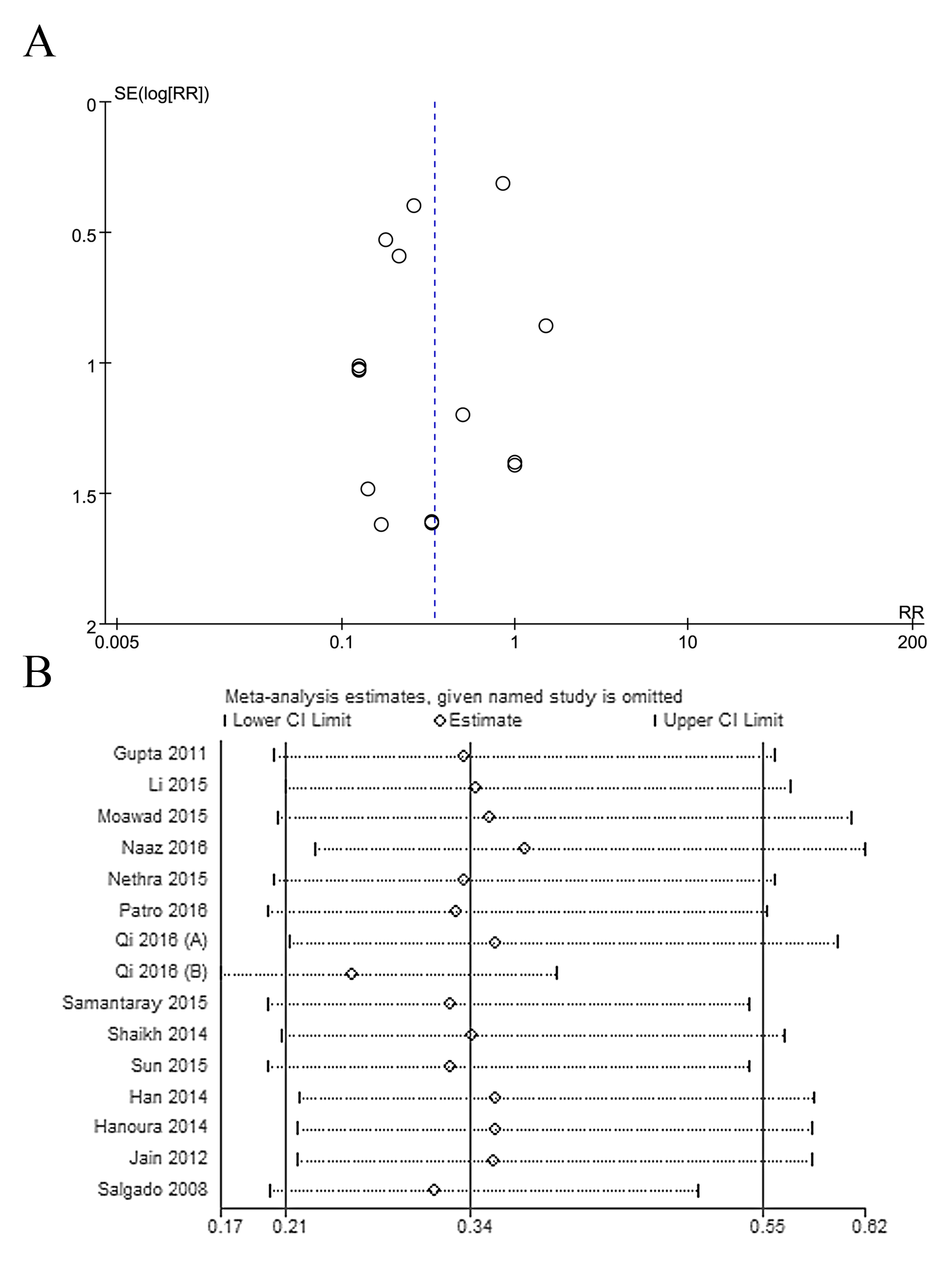

Supplement: S1 Fig — A: funnel plot for publication bias for incidence of shivering, B: sensitivity analysis for the shivering by removing each study individually. (TIF) [file pone.0183154.s001.tif]

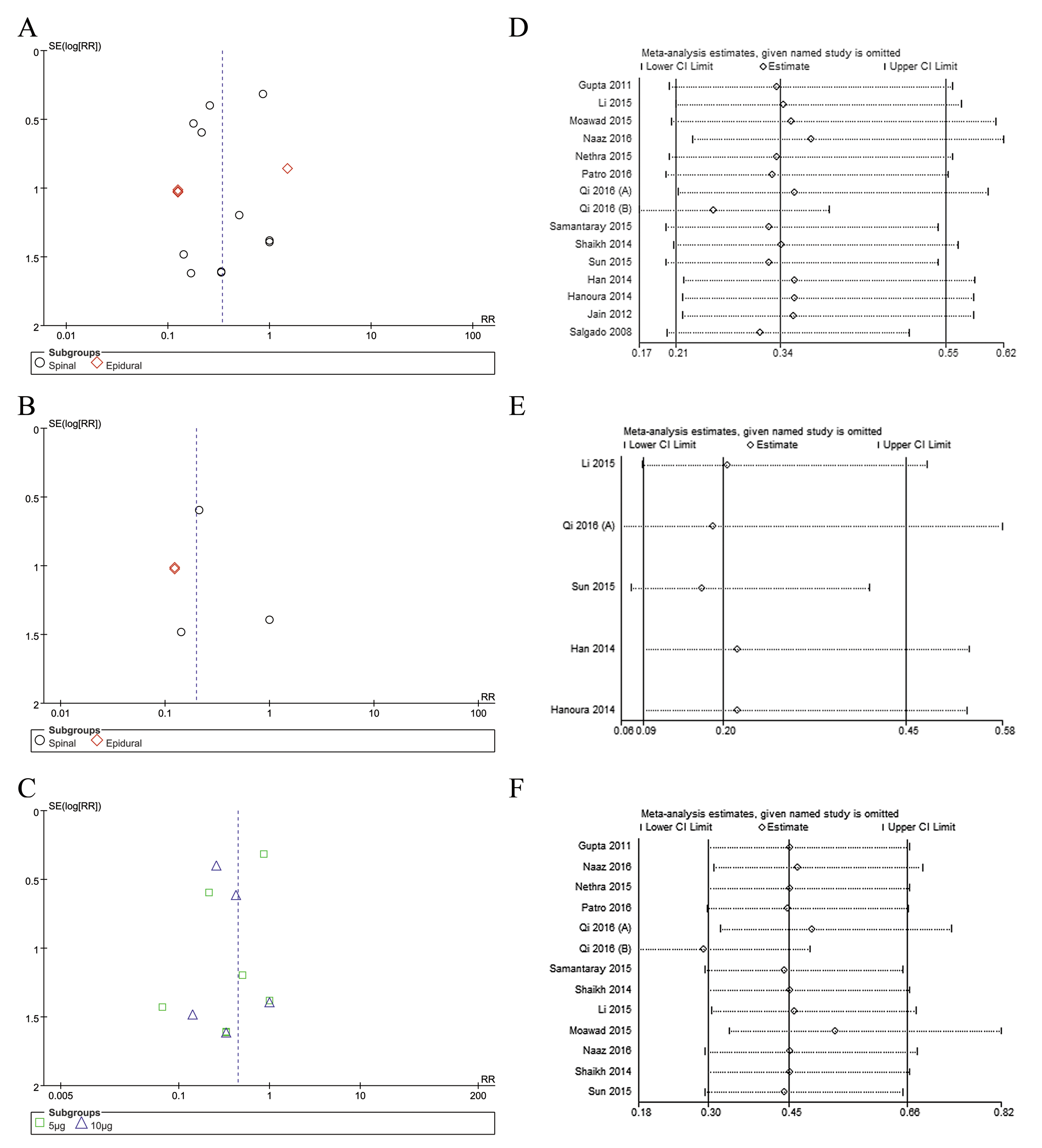

Supplement: S2 Fig — A: funnel plot for publication bias for routes of administration, B: funnel plot for publication bias for cesarean section, C: funnel plot for publication bias for different doses of dexmedetomidine, D: sensitivity analysis for routes of administration, E: sensitivity analysis for cesarean section, F: sensitivity analysis for different doses of dexmedetomidine. (TIF) [file pone.0183154.s002.tif]

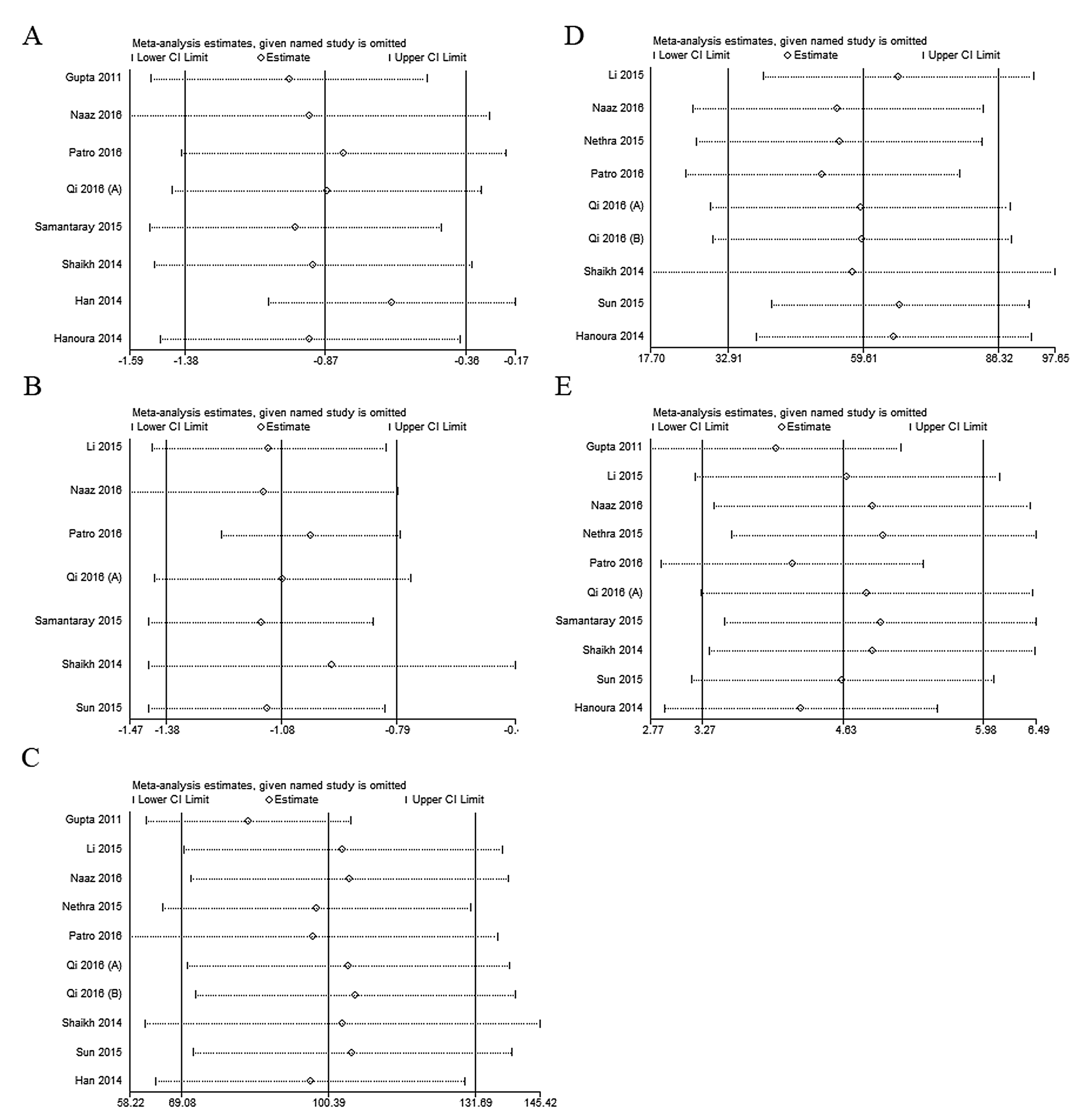

Supplement: S3 Fig — A: sensitivity analysis for onset of sensory block, B: sensitivity analysis for onset of motor block, C: sensitivity analysis for duration of sensory block, D: sensitivity analysis for duration of motor block, E: sensitivity analysis for time to rescue analgesia. (TIF) [file pone.0183154.s003.tif]

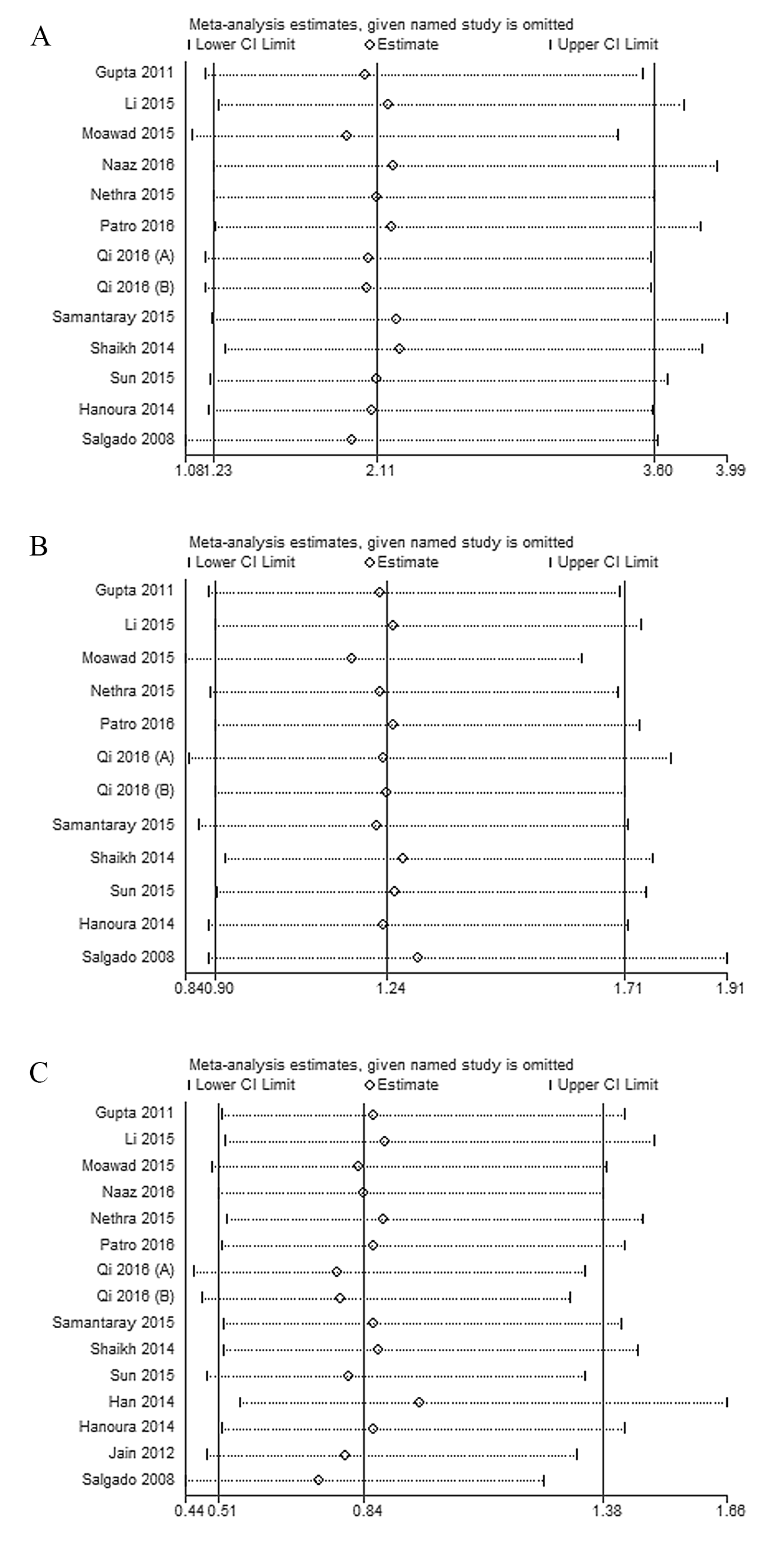

Supplement: S4 Fig — A: sensitivity analysis for bradycardia, B: sensitivity analysis for hypotension, C: sensitivity analysis for nausea/vomiting. (TIF) [file pone.0183154.s004.tif]

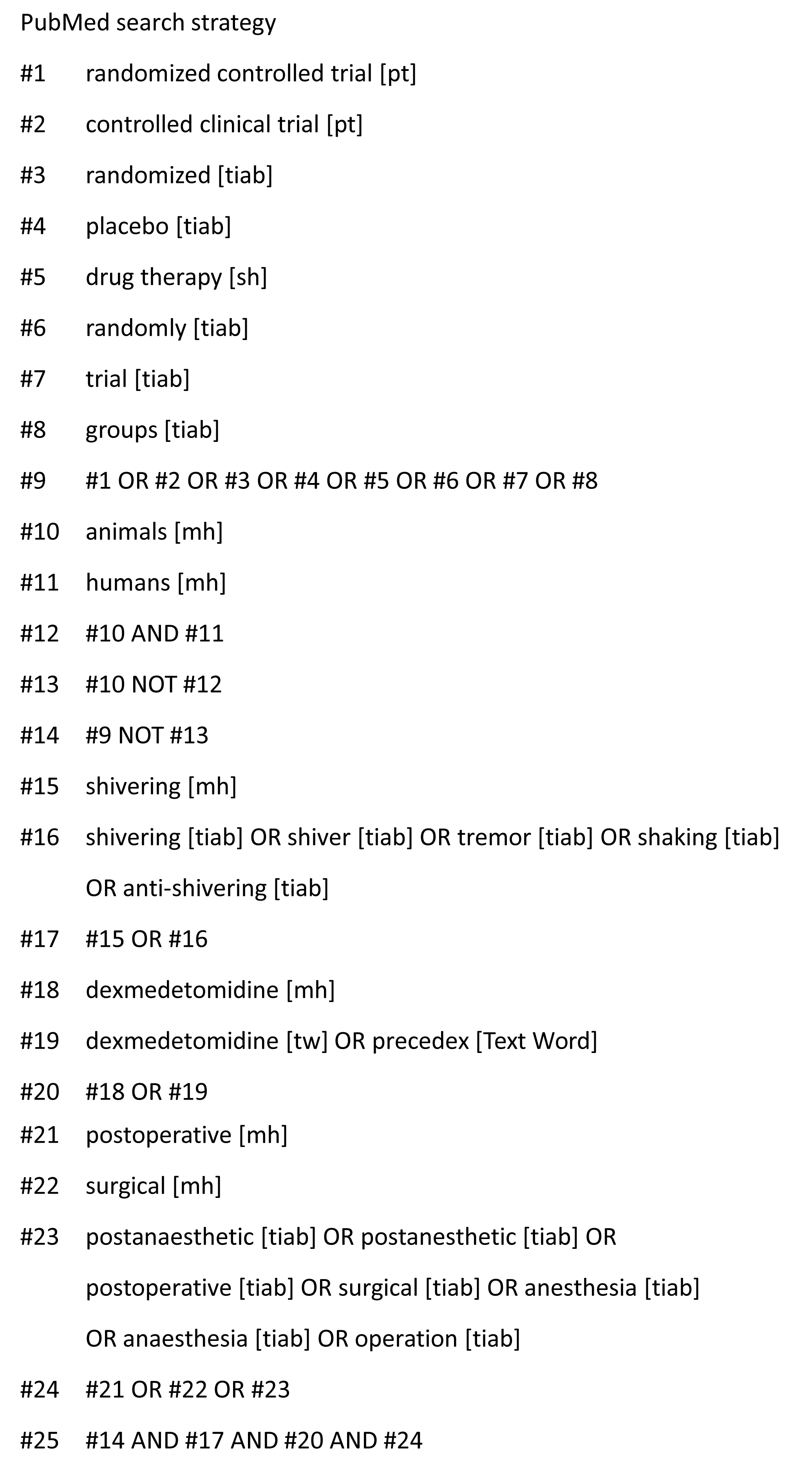

Supplement: S1 Appendix — (TIF) [file pone.0183154.s008.tif]
