# Supplementary material for: Dexmedetomidine as a neuraxial adjuvant for prevention of perioperative shivering: Meta-analysis of randomized controlled trials
Source: PLoS One. 2017 Aug 22;12(8):e0183154. doi: 10.1371/journal.pone.0183154 (PMC5567500; doi:10.1371/journal.pone.0183154)
Supplement: S1 Table — Abbreviations: ASA, American Society of Anesthesiologists physical status; SA, spinal anesthesia; EA, epidural anesthesia; L, Lumbar; DEX, dexmedetomidine. (DOCX) [file pone.0183154.s005.docx]

**Table S1. Characteristics of studies included in the present systematic review and meta-analysis.**

| Study | Location | Jadad score | Surgery | ASA | Route | Position | Patient | Age, mean or median (y) | Time of surgery, mean or median (min) | Comparisons |  |
| --- | --- | --- | --- | --- | --- | --- | --- | --- | --- | --- | --- |
| Gupta 2011 | India | 4 | Lower limb surgery | I−II | SA | L3−4 or L4-5 | 30 | 41.27 | - | DEX 5.0μg | |
|  |  |  |  |  |  |  | 30 | 40.4 | - | Placebo | |
| Moawad 2015 | Egypt | 4 | Transurethral prostatectomy | I−III | SA | L3−4 or L4−5 | 40 | 60.65 | 78.87 | DEX 10.0μg | |
|  |  |  |  |  |  |  | 40 | 59.40 | 79.88 | Placebo | |
| Naaz 2016 | India | 4 | Lower abdominal surgery | I−II | SA | L3−4 | 20 | 39.6 | 94.69 | DEX 5.0μg | |
|  |  |  |  |  |  |  | 20 | 39.8 | 93.77 | DEX 10.0μg | |
|  |  |  |  |  |  |  | 20 | 43.32 | 101.15 | DEX 15.0μg | |
|  |  |  |  |  |  |  | 20 | 45.85 | 108.28 | DEX 20.0μg | |
|  |  |  |  |  |  |  | 20 | 44.21 | 97.5 | Placebo | |
| Nethra 2015 | India | 5 | Perianal surgery | I−II | SA | L3−4 or L4−5 | 20 | 38.56 | 26.25 | DEX 5.0μg | |
|  |  |  |  |  |  |  | 20 | 39.90 | 28.95 | Placebo | |
| Patro 2016 | India | 4 | Infra-umbilical surgery | I−II | SA | L3−4 | 30 | 35.3 | 90 | DEX 5.0μg | |
|  |  |  |  |  |  |  | 30 | 31.7 | 90 | Placebo | |
| Qi 2016 (A) | China | 5 | Cesarean section | I−II | SA | L3−4 | 39 | 29.77 | 40.51 | DEX 5.0μg | |
|  |  |  |  |  |  |  | 40 | 29.63 | 39.45 | Morphine 100μg | |
|  |  |  |  |  |  |  | 39 | 29.74 | 38.41 | Placebo | |
| Qi 2016 (B) | China | 5 | Hysteroscopic surgery | I−II | SA | L2−3 | 36 | 32.67 | 32.44 | DEX 5.0μg | |
|  |  |  |  |  |  |  | 36 | 30.89 | 29.89 | Fentanyl 15.0μg | |
|  |  |  |  |  |  |  | 36 | 30.69 | 30.17 | Placebo | |
| Samantaray 2015 | India | 5 | Endourologic-al procedure | I−II | SA | L3−4 or L4−5 | 20 | 41.3 | 65 | DEX 5.0μg | |
|  |  |  |  |  |  |  | 20 | 47.0 | 63 | Midazolam 1mg | |
|  |  |  |  |  |  |  | 20 | 44.1 | 62 | Placebo | |
| Shaikh 2014 | India | 4 | Infra-umbilical surgery | I−II | SA | L3−4 | 30 | 35.17 | - | DEX 5.0μg | |
|  |  |  |  |  |  |  | 30 | 35.16 | - | DEX 10.0μg | |
|  |  |  |  |  |  |  | 30 | 34.37 | - | Placebo | |
| Li 2015 | China | 4 | Cesarean section | I−II | SA | L2−3 or L3−4 | 21 | 29.09 | 43.31 | DEX 10.0μg | |
|  |  |  |  |  |  |  | 21 | 29.57 | 45.22 | Clonidine 75μg | |
|  |  |  |  |  |  |  | 21 | 30.94 | 44.92 | Fentanyl 15μg | |
|  |  |  |  |  |  |  | 21 | 30.30 | 45.89 | Placebo | |
| Sun 2015 | China | 5 | Cesarean section | I−II | SA | L2−3 or L3−4 | 30 | 28.56 | 43.11 | DEX 10.0μg | |
|  |  |  |  |  |  |  | 30 | 30.55 | 43.2 | Fentanyl 25μg | |
|  |  |  |  |  |  |  | 30 | 29.75 | 42.89 | Placebo | |
| Gupta 2014 | India | 5 | Lower abdominal surgery | I−II | SA | L3−4 | 30 | 46.60 | 95.43 | DEX 5.0μg | |
|  |  |  |  |  |  |  | 30 | 42.60 | 91.67 | Buprenorphine 60μg | |
| Suresh 2016 | India | 4 | Lower abdom- inal surgery | I−II | SA | L3−4 | 30 | 34.93 | - | DEX 5.0μg | |
|  |  |  |  |  |  |  | 30 | 34.93 | - | Fentanyl 25μg | |
| Das 2015 | India | 5 | abdominal hysterectomy | I−II | SA | L3−4 | 50 | 45.2 | 98.2 | DEX 5.0μg | |
|  |  |  |  |  |  |  | 50 | 44.7 | 95 | DEX 10.0μg | |
| Gupta 2016 | India | 5 | Lower abdom- inal and lower limb surgery | I−II | SA | L3−4 | 30 | 43.40 | 100 | DEX 2.5μg | |
|  |  |  |  |  |  |  | 30 | 37.37 | 99.17 | DEX 5.0μg | |
|  |  |  |  |  |  |  | 30 | 41.50 | 100.10 | DEX 10.0μg | |
| Halder 2014 | India | 5 | Lower limb surgery | I−II | SA | L3−4 | 40 | 54.9 | 109 | DEX 5.0μg | |
|  |  |  |  |  |  |  | 40 | 56.3 | 111 | DEX 10.0μg | |
| Han 2014 | China | 5 | Cesarean section | I−II | EA | L2−3 | 20 | 28.8 | 45 | DEX 1μg/kg | |
|  |  |  |  |  |  |  | 20 | 29.3 | 44 | Fentanyl 1μg/kg | |
|  |  |  |  |  |  |  | 20 | 28.4 | 46 | Placebo | |
| Hanoura 2014 | Egypt | 5 | Cesarean section | I−II | EA | L3−4 | 25 | 29.8 | 49 | DEX 1.0μg/kg | |
|  |  |  |  |  |  |  | 25 | 28.8 | 48 | Placebo | |
| Jain 2012 | India | 5 | Lower limb surgery | I−II | EA | - | 30 | 38.87 | 80.9 | DEX 2.0μg/kg | |
|  |  |  |  |  |  |  | 30 | 40 | 79.3 | Placebo | |
| Salgado 2008 | Portugal | 5 | hernia repair or varicose vein surgery | I−II | EA | L3−4 | 20 | 46.5 | 213 | DEX 1.0μg/kg | |
|  |  |  |  |  |  |  | 20 | 44.3 | 208 | Placebo | |
| Bajwa 2011 (A) | India | 4 | lower limb surgery | I−II | EA | L3−4 | 50 | 38.68 | 102.48 | DEX 1.0μg/kg | |
|  |  |  |  |  |  |  | 50 | 34.06 | 108.78 | Fentanyl 1.0μg/kg | |
| Bajwa 2011 (B) | India | 5 | vaginal hysterectomy | I−II | EA | - | 25 | 50.38 | 96.34 | DEX 1.5μg/kg | |
|  |  |  |  |  |  |  | 25 | 52.06 | 99.78 | Clonidine 2.0μg/kg | |
| Fatima 2016 | India | 5 | abdominal hysterectomy | I−II | EA | L2−3 or L3−4 | 30 | 44.13 | - | DEX 1.0μg/kg | |
|  |  |  |  |  |  |  | 30 | 42.86 | - | Butorphanol 10.0μg/kg | |
| Shaikh 2016 | India | 5 | lower limb surgery | I−II | EA | - | 30 | 35.17 | 111.83 | DEX 1.0μg/kg | |
|  |  |  |  |  |  |  | 30 | 33.87 | 112.67 | Clonidine 2.0μg/kg | |

**Abbreviations:** ASA, American Society of Anesthesiologists physical status; SA, spinal anesthesia; EA, epidural anesthesia; L, Lumbar; DEX, dexmedetomidine.
