# Supplementary material for: Dexmedetomidine as a neuraxial adjuvant for prevention of perioperative shivering: Meta-analysis of randomized controlled trials
Source: PLoS One. 2017 Aug 22;12(8):e0183154. doi: 10.1371/journal.pone.0183154 (PMC5567500; doi:10.1371/journal.pone.0183154)
Supplement: S2 Table — Abbreviations: CI, confidence interval; RR, relative risk; NA, not applicable. (DOCX) [file pone.0183154.s006.docx]

**Table S2. Comparison of incidences of shivering between dexmedetomidine and other adjuvants.**

| **Adjuvants** | **Number of studies** | **Incidence of shivering/total number of patients** | | **Fixed-effect model RR (95% CI)** | **Random-effect model RR (95% CI)** |
| --- | --- | --- | --- | --- | --- |
|  |  | **Dexmedetomidine** | **Control** |  |  |
| **Clonidine** | 3 | 3/76 | 3/76 | 1.00 (0.21 to 4.76) | 1.00 [0.19, 5.22] |
| Spinal route | 1 | 0/21 | 0/21 | NA | NA |
| Epidural route | 2 | 3/55 | 3/55 | 1.00 (0.21 to 4.76) | 1.00 [0.19, 5.22] |
| **Fentanyl** | 6 | 16/187 | 22/187 | 0.73 [0.41, 1.31] | 0.69 [0.32, 1.51] |
| Spinal route | 4 | 14/117 | 17/117 | 0.83 [0.44, 1.55] | 0.64 [0.21, 1.98] |
| Epidural route | 2 | 2/70 | 5/70 | 0.40 [0.08, 1.97] | 0.40 [0.08, 1.99] |
| **Morphine** | 1 | 3/39 | 12/40 | 0.26 [0.08, 0.84] | 0.26 [0.08, 0.84] |
| Spinal route | 1 | 3/39 | 12/40 | 0.26 [0.08, 0.84] | 0.26 [0.08, 0.84] |
| Epidural route | - | - | - | - | - |
| **Midazolam** | 1 | 1/20 | 0/20 | 3.00 [0.13, 69.52] | 3.00 [0.13, 69.52] |
| Spinal route | 1 | 1/20 | 0/20 | 3.00 [0.13, 69.52] | 3.00 [0.13, 69.52] |
| Epidural route | - | - | - | - | - |
| **Buprenorphine** | 1 | 5/30 | 2/30 | 2.50 [0.53, 11.89] | 2.50 [0.53, 11.89] |
| Spinal route | 1 | 5/30 | 2/30 | 2.50 [0.53, 11.89] | 2.50 [0.53, 11.89] |
| Epidural route | - | - | - | - | - |
| **Butorphanol** | 1 | 1/30 | 1/30 | 1.00 [0.07, 15.26] | 1.00 [0.07, 15.26] |
| Spinal route | - | - | - | - | - |
| Epidural route | 1 | 1/30 | 1/30 | 1.00 [0.07, 15.26] | 1.00 [0.07, 15.26] |

**Abbreviations:** CI, confidence interval; RR, relative risk; NA, not applicable.
